# *SOS1* gene family in mangrove (*Kandelia obovata*): Genome-wide identification, characterization, and expression analyses under salt and copper stress

**DOI:** 10.1186/s12870-024-05528-0

**Published:** 2024-08-27

**Authors:** Chenjing Shang, Li Sihui, Chunyuan Li, Quaid Hussain, Pengyu Chen, Muhammad Azhar Hussain, Jackson Nkoh Nkoh

**Affiliations:** 1 Guangdong Provincial Key Laboratory for Plant Epigenetics, Shenzhen Engineering Laboratory for Marine Algal Biotechnology, Shenzhen Public Service Platform for Collaborative Innovation of Marine Algae Industry, Guangdong Engineering Research Center for Marine Algal Biotechnology, College of Life Science and Oceanography, Shenzhen University, Shenzhen, 518060 People’s Republic of China; 2 Institute of Deep-Sea Science and Engineering, Chinese Academy of Sciences, Sanya, 572000 China; 3 College of Physics and Optoelectronic Engineering, Shenzhen University, Shenzhen, 518060 People’s Republic of China

**Keywords:** Salt stress, Copper stress, *SOS1* gene family, *Kandelia obovata*, Expression analysis

## Abstract

**Background:**

Salt Overly Sensitive 1 (*SOS1*), a plasma membrane Na^+^/H^+^ exchanger, is essential for plant salt tolerance. Salt damage is a significant abiotic stress that impacts plant species globally. All living organisms require copper (Cu), a necessary micronutrient and a protein cofactor for many biological and physiological processes. High Cu concentrations, however, may result in pollution that inhibits the growth and development of plants. The function and production of mangrove ecosystems are significantly impacted by rising salinity and copper contamination.

**Results:**

A genome-wide analysis and bioinformatics techniques were used in this study to identify 20 *SOS1* genes in the genome of *Kandelia obovata*. Most of the *SOS1* genes were found on the plasma membrane and dispersed over 11 of the 18 chromosomes. Based on phylogenetic analysis, *KoSOS1s* can be categorized into four groups, similar to *Solanum tuberosum*. *Kandelia obovata's SOS1* gene family expanded due to tandem and segmental duplication. These *SOS1* homologs shared similar protein structures, according to the results of the conserved motif analysis. The coding regions of 20 *KoSOS1* genes consist of amino acids ranging from 466 to 1221, while the exons include amino acids ranging from 3 to 23. In addition, we found that the 2.0 kb upstream promoter region of the *KoSOS1s* gene contains several cis-elements associated with phytohormones and stress responses. According to the expression experiments, seven randomly chosen genes experienced up- and down-regulation of their expression levels in response to copper (CuCl_2_) and salt stressors.

**Conclusions:**

For the first time, this work systematically identified *SOS1* genes in *Kandelia obovata*. Our investigations also encompassed physicochemical properties, evolution, and expression patterns, thereby furnishing a theoretical framework for subsequent research endeavours aimed at functionally characterizing the *Kandelia obovata SOS1* genes throughout the life cycle of plants.

**Supplementary Information:**

The online version contains supplementary material available at 10.1186/s12870-024-05528-0.

## Background

Plants are vulnerable to a range of unfavorable environmental circumstances since their life cycle is sessile [1]. Plants naturally thrive in challenging environments [2], and their natural surroundings consist of non-living and living factors that cause stress [3]. Abiotic stressors, such as excessive salinity and heavy metals, are the primary limiting factors that negatively impact plant growth and development. These stressors eventually have a detrimental effect on crop output and sustainability [4, 5]. Salinity is a major contributor to reduced crop yield, negatively impacting plant vitality [1]. A significant amount of arable land experiences increasing Na^+^ concentration ([Na^+^]), making salinity stress a significant agricultural concern [6, 7]. High levels of sodium ions (Na^+^) disturb the balance of ion concentrations within cells and hinder plant metabolism. As sodium (Na^+)^ is unnecessary for plants, any excess amount of sodium ions [Na^+^] should be removed or stored in specific compartments to minimize the harmful effects of excessive ionic toxicity [6, 8].

Nonetheless, the productivity and function of the mangrove ecosystem are strongly impacted by rising salinity and heavy metal contamination [9]. Mangroves possess unique mechanisms to cope with the stress caused by salt exposure. These mechanisms enable them to conduct gaseous exchange, reproduction, and physiological adaptations for salt exclusion and excretion [10]. Certain mangrove species release organic chemicals at the molecular level to control salt content and maintain osmotic balance [11]. Nevertheless, the efficacy of these mechanisms that exclude and excrete salt in the presence of salt and heavy metal stress is still unknown. Gaining insight into these regulatory mechanisms during prolonged stress could enhance our understanding and aid in developing novel management solutions.

For numerous physiological processes, copper is a necessary protein cofactor and is a vital component of all living organisms [12]. Nevertheless, elevated levels of Cu can result in pollution, resulting in a decline in plant growth and hindered developmental processes [13]. As stated by Cano-Gauci and Sarkar [14], the high reactivity of Cu can lead to substantial oxidative harm in cells, impairments in germination, difficulties in flowering, and delayed growth of roots. Eukaryotic organisms have developed a mechanism to efficiently control the uptake and distribution of copper in response to both deficiencies and surpluses of this vital element [15]. Copper plays a crucial role in various physiological processes in plants, including cell wall metabolism, removal of superoxide radicals, photosynthesis, mitochondrial respiration, and detection of ethylene gas [16]. Plants with low amounts of copper have many abnormal characteristics, such as decreased water transportation, impaired growth of young leaves, and slower growth and reproductive development [17]. Shen et al. [18] reported that *Kandelia Obovata* species exhibited different tolerance levels to several heavy metals. It could withstand copper stress at a concentration of 400 mg/L. Hence, the experiment encompassed various concentrations of Cu solution, ranging from 0 to 400 mg/L [19].

The Salt Overly Sensitive (*SOS*) signaling system is crucial for plants to respond to salt stress. The composition comprises three constituents: *SOS1*, *SOS2*, and *SOS3* [20, 21]. *SOS1* is a transporter that moves Na^+^ ions out of the root and into the xylem vessel for long-distance transport [22]. The *SOS1* genes were initially discovered in Arabidopsis [23, 24] and were named *AtNHX1*-*AtNHX8. AtNHX7*, also known as *AtSOS1*, plays a crucial role in the *SOS* signaling system [25]. The *SOS1* protein is in the plasma membrane [26]. *AtSOS1* expression is primarily seen in parenchyma along the xylem-symplast border of the root, stem, and leaf, as well as in epidermal cells at the tip of the root. This implies that this transporter is involved in controlling the long-distance movement of Na^+^ in plants as well as eliminating Na^+^ from the plant into the surrounding medium [27]. With 12 transmembrane domains in each unit's N-terminal region, *SOS1* operates as a homodimer. It also has a long C-terminal region with three domains: an auto-inhibitory domain, a cyclic nucleotide-binding domain, and a cytosolic domain [20, 28]. Plant tolerance to salt is regulated by SOS proteins [20]. The accumulation of Na^+^ in the xylem and shoot decreased as a result of *SOS1* overexpression [26].

The physiological functions of the related *SOS1* genes have also been studied in cash crop plants, including *Solanum tuberosum* L. [20], wheat [29], tuber mustard [21], Arabidopsis [6], maize [30], and *Chenopodium quinoa Willd*. [31]. The *GmSOS1* mutants in soybeans showed a notable buildup of Na^+^ in their roots, leading to an imbalance between Na^+^ and K^+^. This indicates that *GmSOS1* is crucial in maintaining Na^+^ homeostasis and enhancing salt tolerance in soybeans [32]. The *SOS* pathway in maize exhibits a preserved ability to tolerate salt, and its components (*ZmSOS1* and *ZmCBL8*) play a role in regulating Na^+^ and displaying natural variability in salt tolerance. This makes them valuable gene targets to breed salt-tolerant maize [33]. However, its function in *Kandelia obovata* has not yet been studied.

The existence of the *SOS1* gene family in *Kandelia obovata* is not currently supported by published data. As a result, this study represents the first time that *SOS1* genes have been found throughout the whole genome of *Kandelia obovata*. This work discovered, described, and investigated the expression levels of 20 *SOS1* genes in response to CuCl_2_ and salt treatments. To gain more comprehensive knowledge, various bioinformatics methods were used to examine different elements of the evolutionary patterns of *SOS1* genes in *Kandelia obovata*. Numerous features were examined in the investigation, such as subcellular localization, conserved motifs, cis-elements, phylogenetic connections, chromosomal distribution, physicochemical traits, gene structure, synteny and duplication structures, and the expression profiles of *SOS1* homologs. In this study, the *SOS1* family in *Kandelia obovata* is characterized and analyzed with an emphasis on expression. With this study, we hope to lay the theoretical groundwork for future research on how the *SOS1* family in *Kandelia obovata* plants responds to treatments with CuCl_2_ and salt.

## Results

### Genome-wide identification and characterization of *SOS1* Family Members in *Kandelia obovata* genome

*Kandelia obovata's* genome contains 20 *SOS1* genes, much more than the previously reported *SOS1* in Tuber mustard. The statistical results showed that the protein length ranged from 466 (*KoSOS1007616*) to 1221 (*KoSOS1014074*) amino acids; the average length was 735.8. The molecular weight of the SOS1 family ranged from 51.43 (*KoSOS1007616*) to 131.93 (*KoSOS1014074*), with an average of 80.62 kDa. The isoelectric points (pIs) of the *KoSOS1* proteins varied from 5.07 (*KoSOS1014074*) to 9.58 (*KoSOS1014977*), respectively. Instability Index varying from 27.51 (*KoSOS1003260*) to 43.93 (*KoSOS1007616*). The *SOS1* family's average Aliphatic Index ranged from 100.72 to 122.51, while *KoSOS1013784* had the highest and *KoSOS1012320* had the lowest Aliphatic Index. Grand average hydropathy index (GRAVY) values for 20 SOS1s showed a hydrophobic character, ranging from 0.089 (KoSOS1014074) to 0.673 (*KoSOS1012320*). Next, the Na^+^/H^+^ exchanger (NHX) domain content of the candidates was verified using the SMART program (Table 1). Determining the subcellular location of *SOS1* proteins would facilitate a better understanding of the molecular function. Twenty *SOS1s* were most likely located in the plasma membrane, lysosomal, vacuolar, golgi, endoplasmic reticulum, and peroxisomal compartments, based on the subcellular localization prediction of *SOS1* proteins (Table 1, S1).

**Table 1:** Detailed information on the *SOS1* gene family was identified in *Kandelia obovata*

*Name*	Gene ID	Size (AA)	MW(KDa)	pI	Instability Index	Aliphatic Index	GRAVY	Na + /H + Exchanger Domain (start–end)
*KoSOS1000168*	GWHGACBH000168.1	485	53.75	6.70	38.59	110.68	0.496	25–137;178–385
*KoSOS1000949*	GWHGACBH000949.1	731	79.70	9.34	34.41	109.64	0.357	30–432
*KoSOS1002250*	GWHGACBH002250.1	935	101.89	8.41	40.50	108.66	0.275	155–546
*KoSOS1002689*	GWHGACBH002689.1	519	57.42	8.65	43.65	107.05	0.532	22–442
*KoSOS1003260*	GWHGACBH003260.1	549	59.66	6.03	27.51	117.67	0.591	168–525
*KoSOS1003751*	GWHGACBH003751.1	821	90.80	9.21	40.58	111.75	0.294	51–446
*KoSOS1004234*	GWHGACBH004234.1	560	60.25	5.27	29.29	114.20	0.551	154–513
*KoSOS1006610*	GWHGACBH006610.1	808	87.24	5.52	40.28	114.64	0.366	119–506
*KoSOS1007241*	GWHGACBH007241.1	541	59.47	6.89	39.37	110.06	0.56	25–444
*KoSOS1007260*	GWHGACBH007260.1	552	61.21	6.23	37.20	108.97	0.528	24–455
*KoSOS1007616*	GWHGACBH007616.1	466	51.43	5.21	43.93	102.10	0.298	31–212
*KoSOS1008568*	GWHGACBH008568.1	1054	118.40	6.52	32.51	110.72	0.373	22–227;199–403
*KoSOS1009308*	GWHGACBH009308.1	471	51.85	5.42	43.83	102.51	0.418	118–235;228–375
*KoSOS1012320*	GWHGACBH012320.1	610	66.32	5.53	28.06	122.51	0.673	191–561
*KoSOS1013173*	GWHGACBH013173.1	805	86.81	7.85	40.89	111.53	0.427	31–426
*KoSOS1013174*	GWHGACBH013174.1	800	86.12	7.22	40.24	111.62	0.425	36–426
*KoSOS1014074*	GWHGACBH013784.1	1221	131.93	5.07	42	104.65	0.089	25–424
*KoSOS1014977*	GWHGACBH014074.1	518	57.07	9.58	42.16	113.15	0.563	33–439
*KoSOS1015221*	GWHGACBH014977.1	1146	126.76	6.24	39.83	101.60	0.108	10–416
*KoSOS1013784*	GWHGACBH015221.1	1124	124.39	6.83	43.10	100.72	0.099	621–995

*AA*^*1*^ Number of amino acids, *Chains*^*2*^ Positive or negative chains, *MW*^*3*^ Molecular weight, *pI*^*4*^ Isoelectric point, *GRAVY*^*5*^ Grand average of hydropathicity

### Chromosomal location of the *SOS1* genes in *Kandelia obovata* genome

**Fig. 1:**
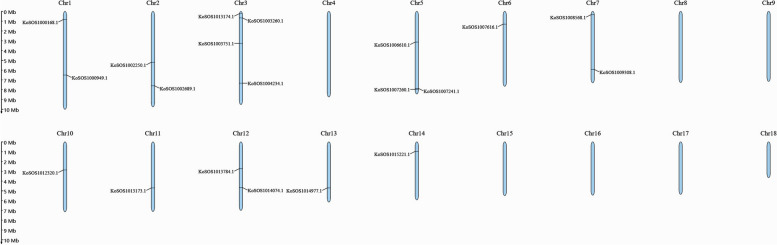
Schematic diagram of the chromosomal location of *Kandelia obovata's SOS1* gene family. Twenty identified *SOS1* homologs genes were mapped to the 11 of 18 chromosomes. The chromosome name is at the top of each bar. The scale of the chromosome is in millions of bases (Mb)

The genomic chromosomal distribution of the discovered *SOS1* genes in *Kandelia obovata* was mapped to the relevant chromosomes using the MapGene2Chromosome (MG2C) program based on the genes' chromosomal locations. The 20 *SOS1* genes were distributed in 11 of the 18 chromosomes of *Kandelia obovata*. Chromosome 3 (Chr3) had the largest *SOS1* genes at four, while chr5 had only three *SOS1* genes. Each chromosome Chr6, Chr10, Chr11, Chr13, and Chr14 contained one gene, and each of the chromosomes Chr1, Chr2, Chr7 and Chr12 contained two genes (Fig. 1, Table S2). This result was in line with a previous study that analyzed repeated events in wheat, maize, potato and other species, indicating that some *SOS1* family members were most likely derived from repetitive events.

### Three-dimensional and transmembrane structure prediction

**Fig. 2:**
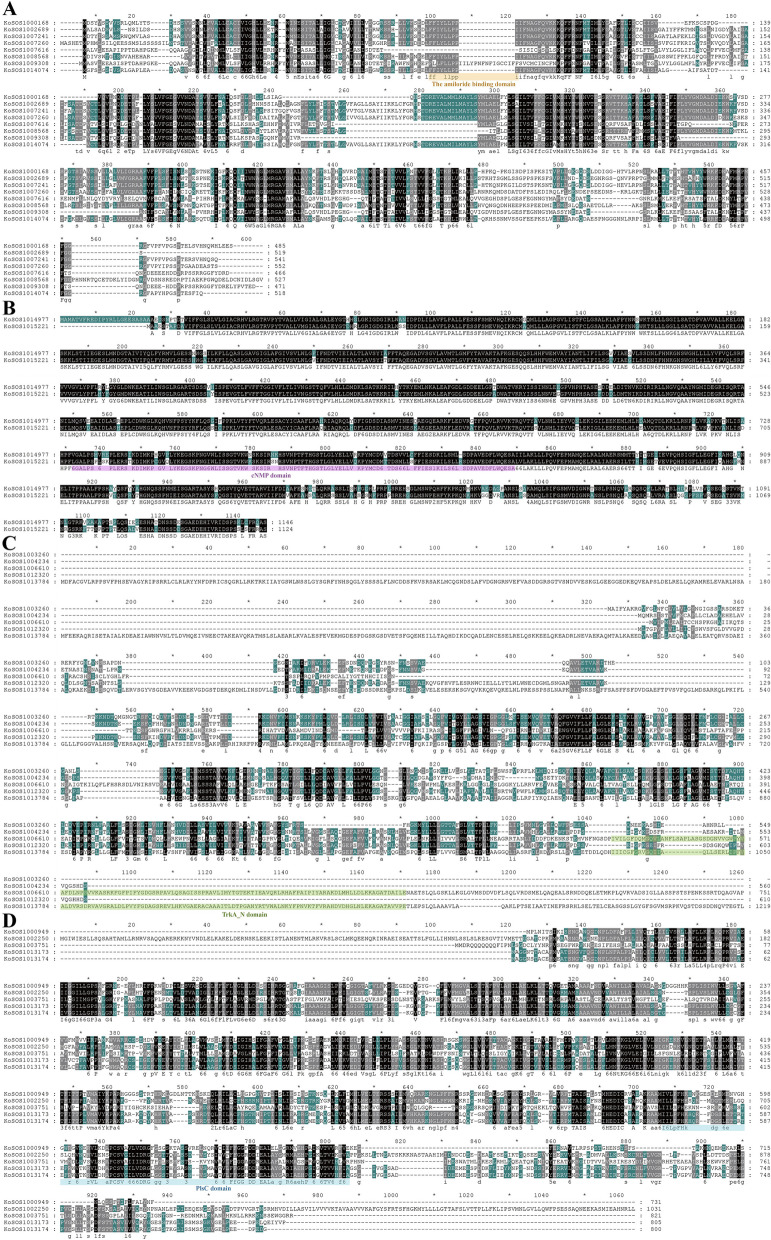
The amino acid sequence has undergone multiple alignments utilizing data derived from each *KoSOS1* gene. Sequence identity and similarity were represented by black, green, and grey letters, respectively. The asterisks (*) above the sequence represent every 10 amino acid residues

**Fig. 3:**
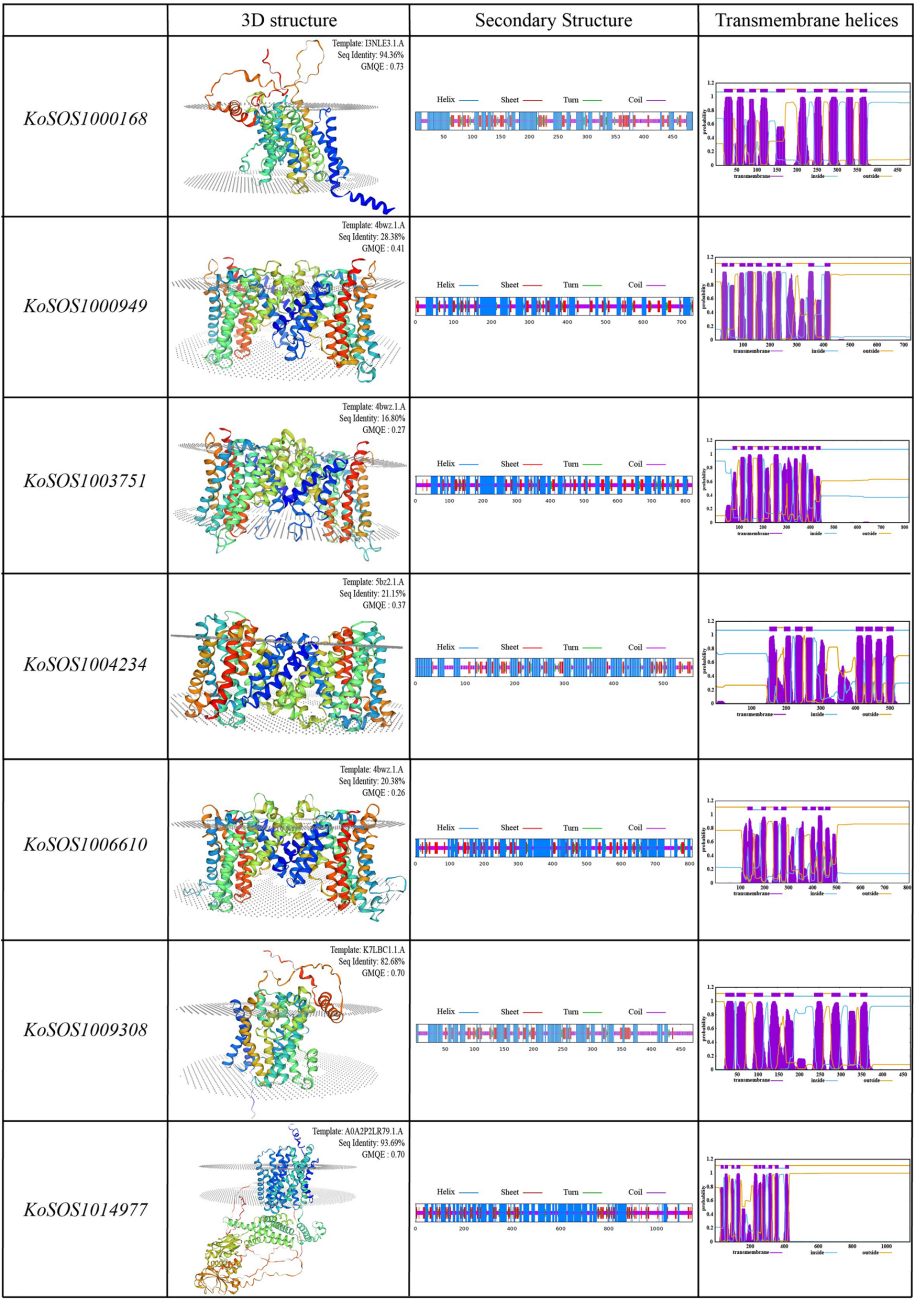
The KoSOS1s exhibit both 3D and transmembrane structures. SWISS-MODEL generates 3D structural homology models. The SOSUI tool has verified the presence of transmembrane structures

Using Clustalw, the protein sequences of the SOS1 homologs were aligned. Comparing homologs from the same subfamily, the study showed that their protein sequences were conserved (Fig. 2). Three transmembrane domains (plsC, TrkA, and cNMP) were shown to be present in SOS1s by domain analysis (PF0099) of *Kandelia obovata* (Fig. 2). The SOPMA/prabi tool and the SWISS-MODEL workspace were used to confirm the protein structures of KoSOS1s (Fig. 3). The results of the domain analysis indicated that the KoSOS1013174 had the plsC domain, the KoSOS1006610, and KoSOS1013784 contained the TrkA domain, the KoSOS1014977 and KoSOS1015221 contained the cNMP domain, and the KoSOS1014074 the amiloride binding domain. According to the findings, the KoSOS1 homologs may respond to abiotic stress by performing conserved tasks. The KoSOS1 proteins were accurately simulated using the templates I3NLE3.1.A (KoSOS1000168), 4bwz.1.A (KoSOS1000949, KoSOSI003751, and KoSOS1006610), 5bz2.1.A (KoSOS1004234), K7LBC1.1.A (KoSOS1009308), and A0A2P2LR79.1.A (KoSOSI014977) as depicted in Fig. 3. The range of sequence identity observed in this study varied from 16.80% to 94.36%. The maximum sequence identity was observed in KoSOS1000168, and the minimum was observed in KoSOSI003751. The values of GMQE ranged from 0.26 to 0.73. The results of this investigation show that the 3D model predictions for KoSOS1 proteins are quite accurate and show the existence of helix and strand structures. As shown in Fig. 3, the secondary structures of the SOS1 proteins from *Kandelia obovata* are similar to those seen in SOS1 proteins from other species.

### SOS1 protein phylogenetic relationships

**Fig. 4:**
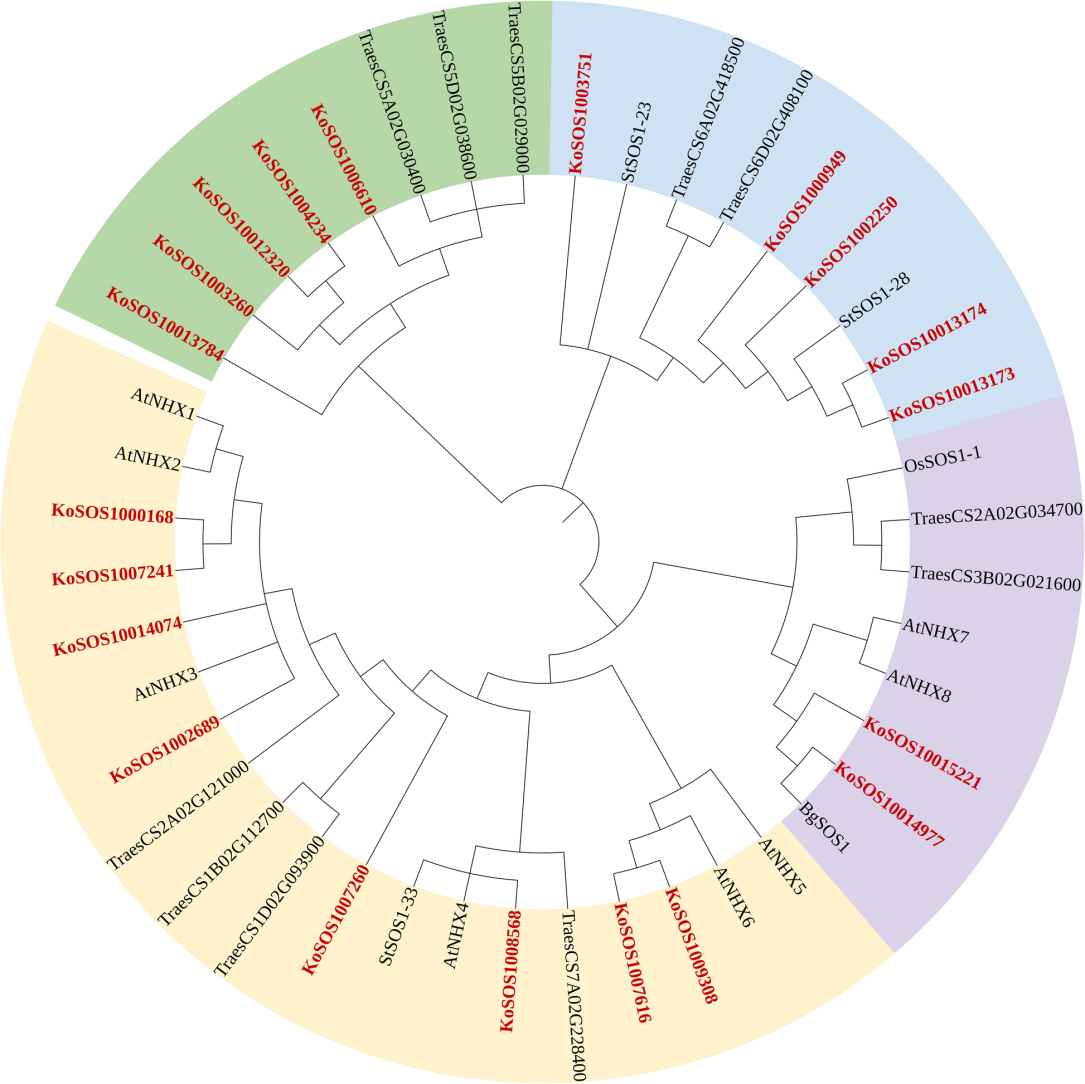
A phylogenetic analysis of SOS1 proteins from *Kandelia obovata* (Ko), *Arabidopsis thaliana* (At), *Triticum aestivum* L. (Traes), *Solanum tuberosum* (St), *Oryza sativa* (Os), and *Bruguiera gymnorhiza* (Bg) was carried out using the maximum likelihood method. The SOS1 proteins were clustered into four groups, i.e., group 1 (orange), group 2 (light green), group 3 (light blue), and group 4 (light pink), each represented by a different colour

To characterize the phylogenetic relationships among SOS1 proteins from *Kandelia obovata* (Ko), *Arabidopsis thaliana* (At), *Triticum aestivum* L. (Traes), *Solanum tuberosum* (St), *Oryza sativa* (Os), and *Bruguiera gymnorhiza* (Bg), an unrooted NJ tree was constructed. A total of 44 SOS1 proteins were used, including 20, 8, 11, 3, 1, and 1 from *Kandelia obovata* (Ko), *Arabidopsis thaliana* (At), *Triticum aestivum* L. (Traes), *Solanum tuberosum* (St), *Oryza sativa* (Os), and *Bruguiera gymnorhiza* (Bg), respectively. The SOS1 proteins were clustered into four groups, i.e., group 1 (orange), group 2 (light green), group 3 (light blue), and group 4 (light pink) (Fig. 4). Group 1 contained eight genes and had the most members (42.11%). Group 2 and Group 3 had 5 5 members (26.32%), and Group 4 had two members (10.53%), respectively. According to the phylogenetic tree, SOS1 proteins could be classified into four groups: group 1 included eight KoSOS1 proteins (KoSOS1000168, KoSOS1007241, KoSOS10014074, KoSOS1002689, KoSOS1007260, KOSOS1008568, KoSOS1007616, and KoSOS1009308), Six AtNHX proteins (AtNHX1-6), four Traes proteins (TraesCS2A02G121000, TraesCS1B02G112700, TraesCS1D02G093900, and TraesCS7A02G228400), and one StSOS1 proteins (StSOS1-33). Group 2 included five KoSOS1 proteins (KoSOS1006610, KoSOS1004234, KoSOS1012320, KoSOS1003260, and KoSOS1013784), three Traes proteins (TraesCS5B02G029000, TraesCS5D02G038600, and TraesCS5A02G030400). The group 3 comprises five KoSOS1 proteins (KoSOS1003751, KoSOS1000949, KoSOS1002250, KoSOS1013173, and KoSOS1013174), two Traes proteins (TraesCS6A02G418500, and TraesCS6D02G408100), two StSOS1 proteins (StSOS1-23, and StSOS1-28). Group 4 included two KoSOS1 proteins (KoSOS1015221 and KoSOS1014977), two AtNHX proteins (AtNHX7/8), two Traes proteins (TraesCS2A02G034700 and TraesCS3B02G021600), one OsSOS1 protein (OsSOS1-1), and one BgSOS1 protein (BgSOS1-1). The phylogenetic relationships indicate that the SOS1 proteins in the *Kandelia obovata* are more strongly homologous to *Triticum aestivum* L., *Solanum tuberosum*, and *Arabidopsis thaliana* than to *Oryza sativa*, and *Bruguiera gymnorhiza*.

### Gene structure and conserved motifs of *KoSOS1* gene family

**Fig. 5:**
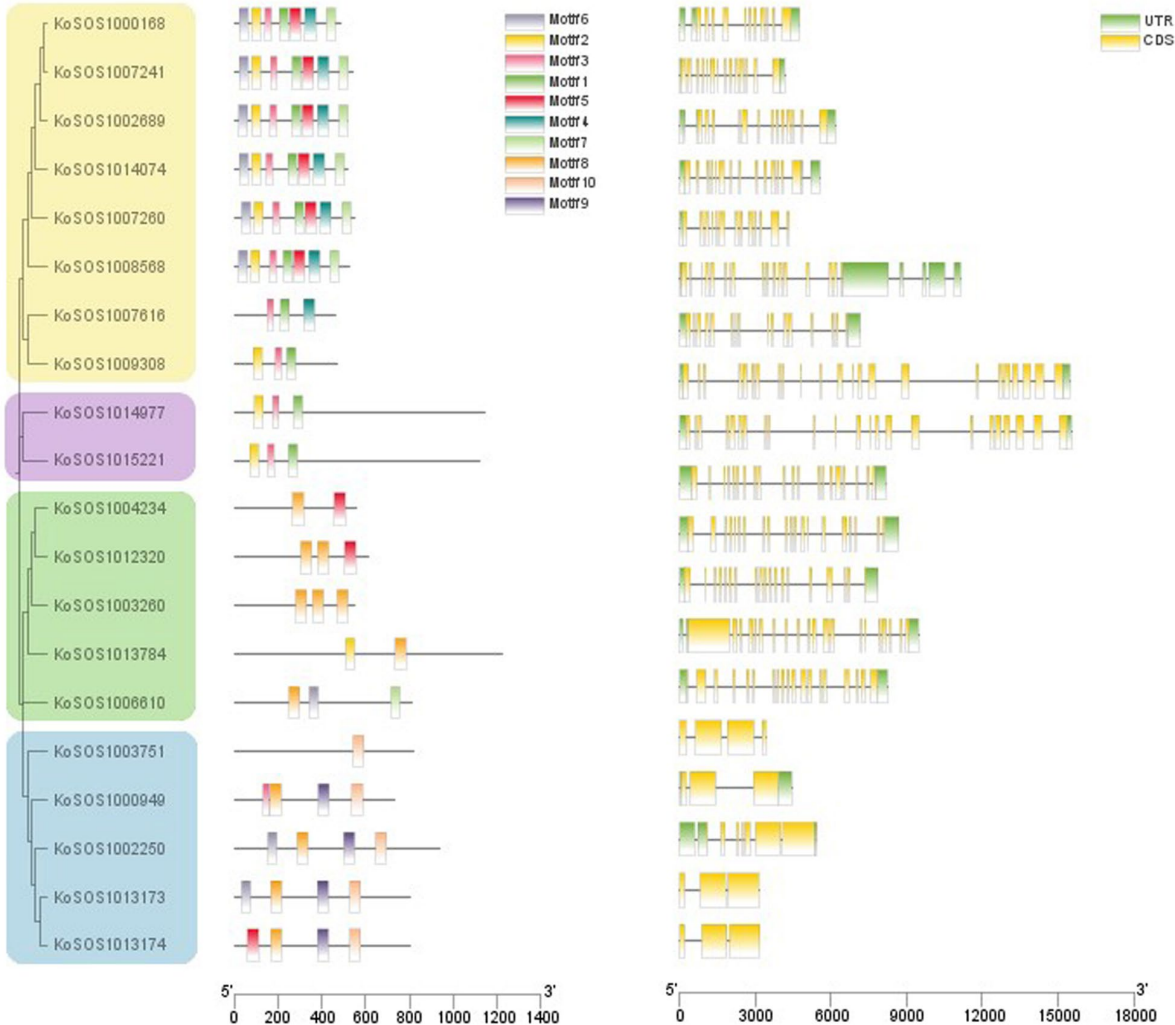
Investigations were conducted into the gene structure and motif makeup of the *SOS1* family genes in *Kandelia obovata*. The *SOS1* genes found in both genomes were classified into four unique groups according to their phylogenetic links, with a specific emphasis on the gene structure of the *SOS1s*. The UTR sections are represented visually as green, while the CDS or exons are displayed in yellow. Introns are identified by a black horizontal line. Moreover, the preserved patterns in the SOS1s are identified by a distinct letter. Colorful boxes with distinct motifs are displayed

To gain a deeper comprehension of the correlation between the structure and function of these KoSOS1 proteins, an analysis of gene structure and conserved motifs was conducted to create separate phylogenies. A phylogenetic tree was constructed utilizing the individual sequences of the SOS1 protein. The SOS1 proteins were categorized into four distinct groups, and this tree represented the evolutionary groups described before. The study aimed to examine the exon–intron patterns of *SOS1* genes in order to investigate gene expansion in the *Kandelia obovata* family. Through the analysis of exon–intron structures and conserved motifs, as illustrated in Fig. 5, we discovered that the *SOS1* gene exhibits different numbers of exons (ranging from 3 to 23) and introns (ranging from 0 to 6).

The number of introns of almost 60% of *KoSOS1* genes was 2, while the remaining *KoSOS1* genes exhibited some introns of 0 (15%), 3 (20%), and 6 (5%). The gene family known as *SOS1s* exhibits a diverse range of gene structures, with most *SOS1* genes containing two UTR/introns. The highest count of exons identified in *KoSOS1014977* and *KoSOS1015221* was 23, respectively. *KoSOS1013173*, and *KoSOS1013174*, *KoSOS1000949* have only three exons, while *KoSOS1003751* have four exons, respectively. The results of the other six *KoSOS1* genes showed that the number of exons ranged from 11 to 20, as shown in Fig. 5. The study revealed that the *SOS1* genes in *Kandelia obovata* have a remarkably preserved gene structure, indicating a significant resemblance to their counterparts in closely related species.

To further investigate the diversity of changes in the *KoSOS1* family during evolution, the conserved motifs of the 20 *KoSOS1s* proteins were analyzed using MEME online software, and ten different conserved motifs (named motifs 1–10) were identified (Fig. 5). The conserved motifs seen in all *SOS1* genes exhibited a range of one to seven; similarly, the *KoSOS1* genes displayed motifs ranging from four to 12. The results showed that motif 8 has been detected in 12 proteins. Motifs 3 and 1 were identified in 11 and 10 proteins, and motifs 2, 5, and 6 were identified in nine proteins, while motifs 4 and 7 were identified in seven. Similarly, motifs 9 and 10 were observed in four and five proteins. We also found that motifs 1–7 were primarily distributed in Group 1, motifs 1–3 in Group 2, and motifs 5 and 8 in Group 3, whereas motifs 8–10 were mainly distributed among Group 4. This study's results indicate a notable level of similarity in the gene structure and amino acid sequence across individuals belonging to the same subfamily of *KoSOS1s*.

### Prediction of cis-elements in the promoter sequences of *KoSOS1* genes

**Fig. 6:**
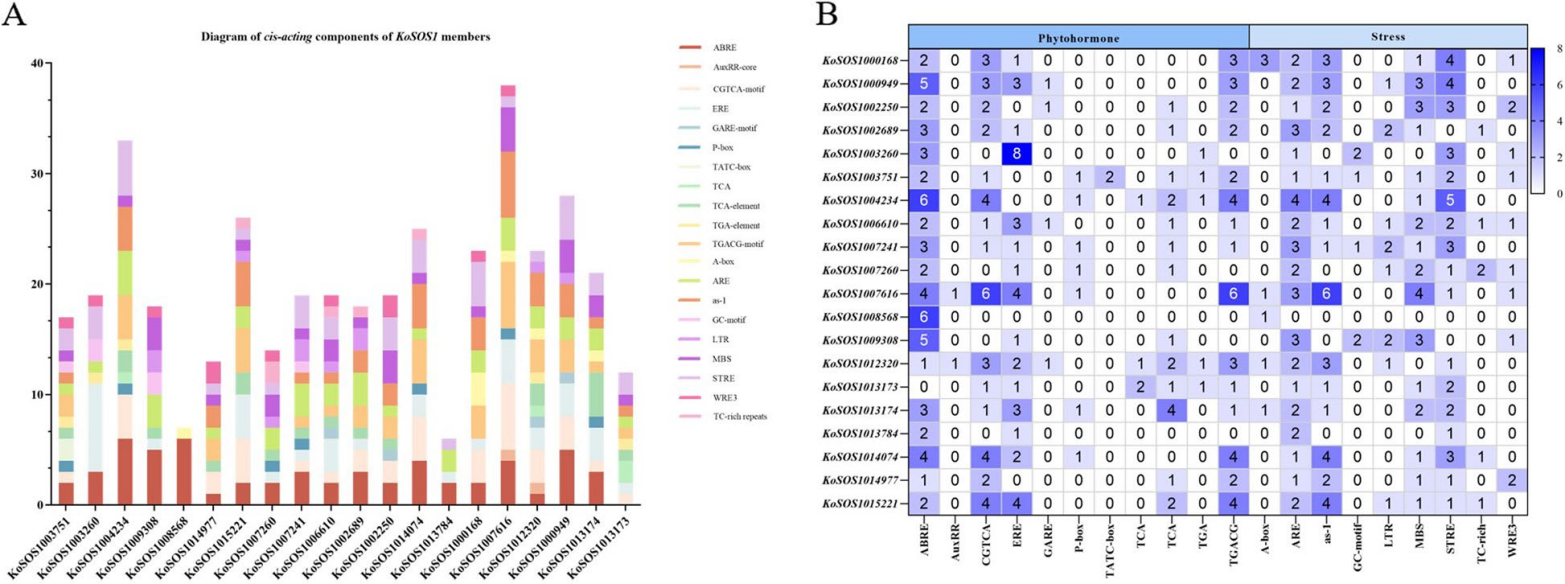
The *KoSOS1* gene promoters contain CREs, or regulatory elements. Twenty *KoSOS1* genes with diverse functions in plant growth, defense, stress response, and hormone response are represented graphically in (A). The predicted CREs' positional distribution on the *KoSOS1* promoters is shown by vertical bars. In this myth, each hue symbolized distinct cis-elements. (B) The number of cis-elements was indicated by different colors. Numbers represent the number of cis-elements, while darker colors imply higher occurrence frequencies

To clarify which hormonal, environmental stress, or developmental-related signal elements are involved in these *KoSOS1s*, we performed a promoter analysis using the PlantCARE server. A large number of basic components were discovered in the upstream sequence (2000 bp) regions, including ABRE, AuxRR-core, CGTCA-motif, ERE, GARE-motif, P-box, TATC-box, TCA, TCA-element, TGA-element, and TGACG-motif were hormonal response-related elements; A-box development-related elements, ARE, as-1, GC-motif, LTR, MBS, STRE, TC-rich repeats, and WRE3 environmental stress-related components (Fig. 6A, B, Table S3). In addition, 214 cis-elements involved in phytohormones (i.e., ABRE (58), AuxRR-core (2), CGTCA-motif (38), ERE (36), GARE-motif (4), P-box (7), TATC-box (2), TCA (4), TCA-element (19), TGA-element (5), and TGACG-motif (39), responses were also identified in the promoter sequences of SOS1 genes (Fig. 6). While 184 cis-elements involved in environmental stress-related components, including A-box (7), ARE (38), as-1 (38), GC-motif (6), LTR (11), MBS (28), STRE (39), TC-rich repeats (6), and WRE3 (11). The variation in the response components demonstrated the regulatory functions of SOS1 genes in numerous physiological and biological processes.

### Synteny and Duplication Analysis of the *KoSOS1* Family

**Fig. 7:**
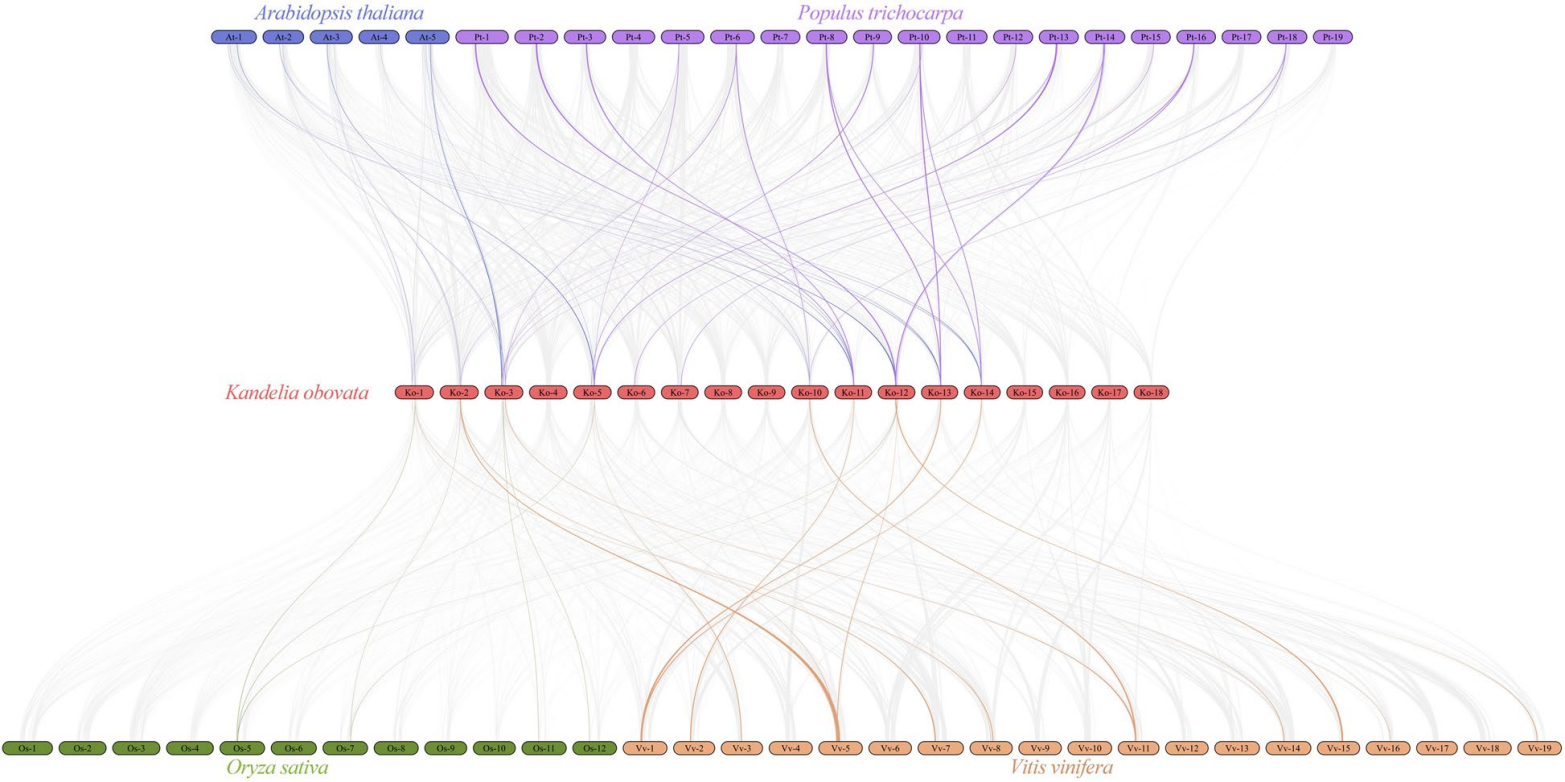
*SOS1* gene synteny study in the chromosomes of *Vitis vinifera*, *Oryza sativa*, *Populus trichocarpa*, *Arabidopsis thaliana*, and *Kandelia obovata*. *Kandelia obovata's* and the other four plant species' genomes' collinear blocks are highlighted by the other colored lines, while the background's grey lines draw attention to the syntenic *SOS1* gene pairs. The box's many colors stood for different kinds of plants

According to a synteny analysis, *Kandelia obovata* (Ko) and the other four inherited plant species, including *Arabidopsis thaliana* (At), *Populus trichocarpa* (Pt), *Oryza sativa* (Os), and *Vitis vinifera* (Vv) all have substantial orthologs of the SOS1 genes (Fig. 7). The *KoSOS1* genes were unevenly distributed in different chromosomes, and some chromosomes have more *KoSOS1* genes compared to others, with numbers ranging from 1 to 4 in each chromosome. Briefly, in chromosome 1, two genes of Ko displayed syntenic associations with two At-3/5, two Pt-5/13, one Os-5, and three Vv-5/8/14 chromosomes. In chromosome 2, two genes of Ko showed a collinear relationship with two At-3, two Pt-13/14, one Os-7, and two Vv-5/7 chromosomes. On the other hand, in chromosome 3, four genes of Ko showed a collinear relationship with five At-2 (2) and At5 (3), six Pt-6/10/12/14/15/18, two Os-11/12, and two Vv-8/18 chromosomes. In chromosome 5, three genes of Ko displayed syntenic associations with two At-3/5, four Pt-5/9/13/14, one Os-5, and three Vv-3/5/14 chromosomes. In chromosome 6, one gene of Ko showed a collinear relationship with one Pt16 chromosome, while in chromosome 7, two genes of Ko showed a syntenic relationship with one At-5, one Pt-16, and one Vv-19 chromosome. Similarly, each one gene of Ko displayed syntenic associations with chromosome 10 (two At-2/5, two Pt-6/18, and one Vv-11), chromosome 11 (two At-1/5, two Pt-1/3, and one Vv-2), chromosome 13 (two At-1/4, two Pt-8/10, and one Vv-1), and chromosome 14 (two At-2, two Pt-8/10, and one Vv-1). In chromosome 12, two genes of Ko showed a collinear relationship with one At-1, two Pt-2/14, two Os-5/7, and two Vv-5/15 chromosomes. The *KoSOS1s* gene family was largely shaped by segmental repetition and whole-genome duplication, as evidenced by the survival of several homologs of the genus *Kandelia obovata* (*KoSOS1s*) in syntenic associations with *Arabidopsis thaliana*, *Populus trichocarpa*, *Oryza sativa*, and *Vitis vinifera*.

**Fig. 8:**
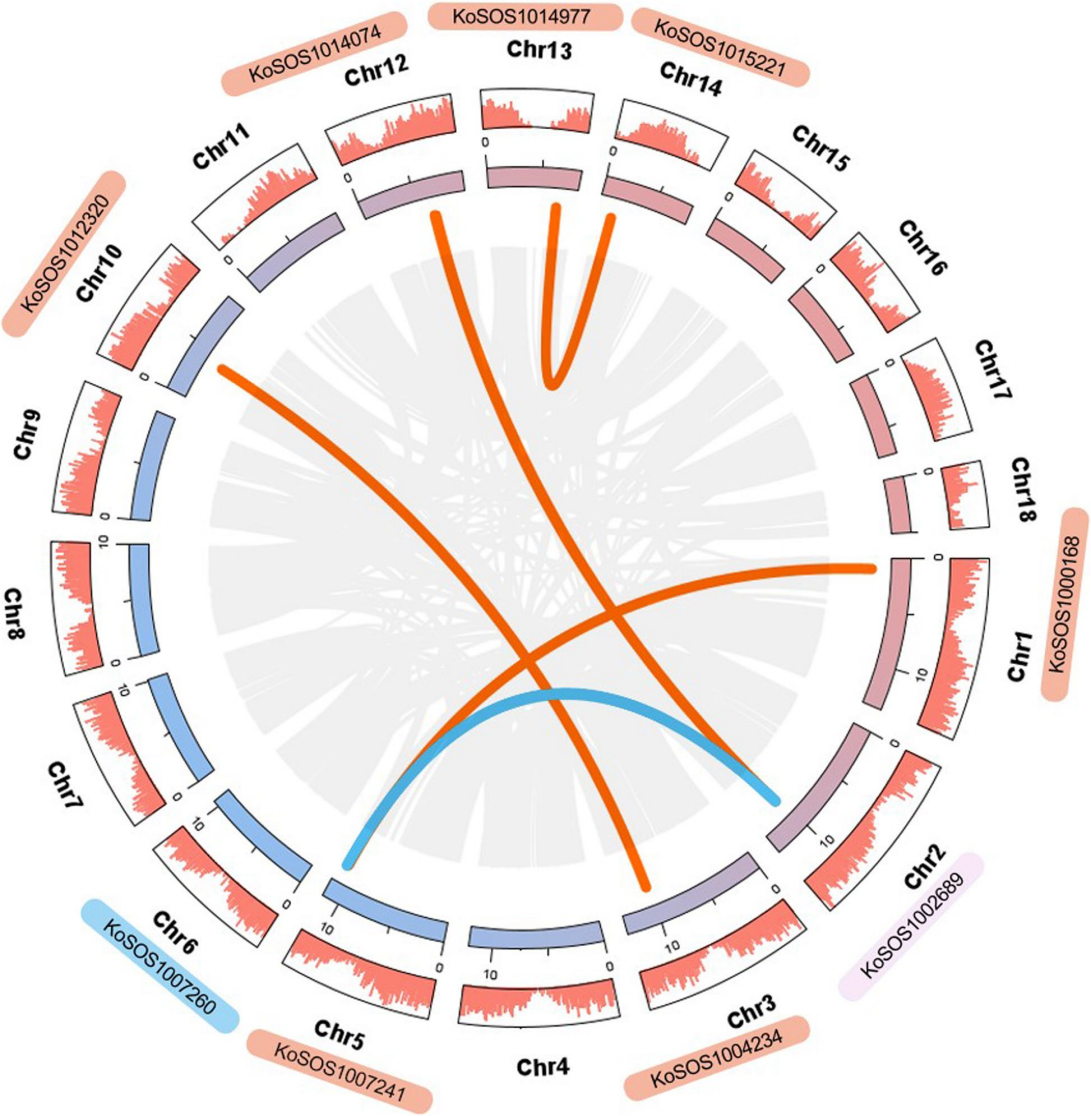
The *KoSOS1* gene's chromosomal distribution and interchromosomal connections are shown as circles. The syntenic blocks in the genome of *Kandelia obovata* are shown by the grey lines in the background, while the red and blue lines represent the syntenic *SOS1* gene pair

Multiple homologs of *Kandelia obovata*, known as *KoSOS1s*, have consistently coexisted with *Arabidopsis thaliana*, *Solanum tuberosum*, *Populus trichocarpa*, *Vitis vinifera*, and *Oryza sativa* through a syntenic relationship. This discovery indicates that both the repeating of segments and the duplication of the entire genome were important factors in the evolution of the *KoSOS1s* gene family. New gene families and plant genomes are encouraged to evolve through segmental and tandem duplication. To gain a deeper understanding of the duplication activities of the *Kandelia obovata SOS1* gene, we conducted an investigation into the segmental and tandem duplications within the *KoSOS1* gene family. The chromosomal dispersals of nine *KoSOS1* genes were evaluated. Five sets of duplicated genes were found, as shown in Fig. 8. The investigation revealed a single instance of segmental duplication involving the gene pairs *KoSOS1000168* and *KoSOS1007241*, *KoSOS1002689* and *KoSOS1007260*, *KoSOS1002689* and *KoSOS1014074*, *KoSOS1004234* and *KoSOS1012320*, and *KoSOS1014977* and *KoSOS1015221* on chromosomes Chr1 and Chr5, Chr2 and Chr6, Chr2 and Chr12, Chr3 and Chr10, and Chr13 and Chr14. Significantly, the *KoSOS1* gene was not present on the other chromosomes.

A thorough investigation was conducted to determine the Ka (non-synonymous substitution rate), Ks (synonymous substitution rate), and the Ka/Ks ratio for the *Kandelia obovata SOS1* gene family, with the aim of enhancing our understanding of the evolutionary limitations that impact it. The duplicated gene pairs of *KoSOS1s* showed Ka/Ks ratios of 1.21, 1.03, 0.79, 1.56, and 0.88. This indicates that the *SOS1* gene family in *Kandelia obovata* may have experienced selective pressures or a discriminating load during their evolutionary history (Table 2).

**Table 2:** Detailed information on the Ka, Ks, and Ka/Ks ratio in *Kandelia obovata*

Name	Method	Ka	Ks	Ka/Ks	Duplicated Type
KoSOS1000168 and KoSOS1007241	NG	1.28	1.06	1.21	WGD or Segmental
KoSOS1002689 and KoSOS1007260	NG	1.23	1.20	1.03	
KoSOS1002689 and KoSOS1014074	NG	0.96	1.21	0.79	
KoSOS1004234 and KoSOS1012320	NG	1.25	0.8	1.56	
KoSOS1014977 and KoSOS1015221	NG	1.21	1.38	0.88	

### Expression analysis of *SOS1* genes *Kandelia obovata* leaves under salt stress

**Fig. 9:**
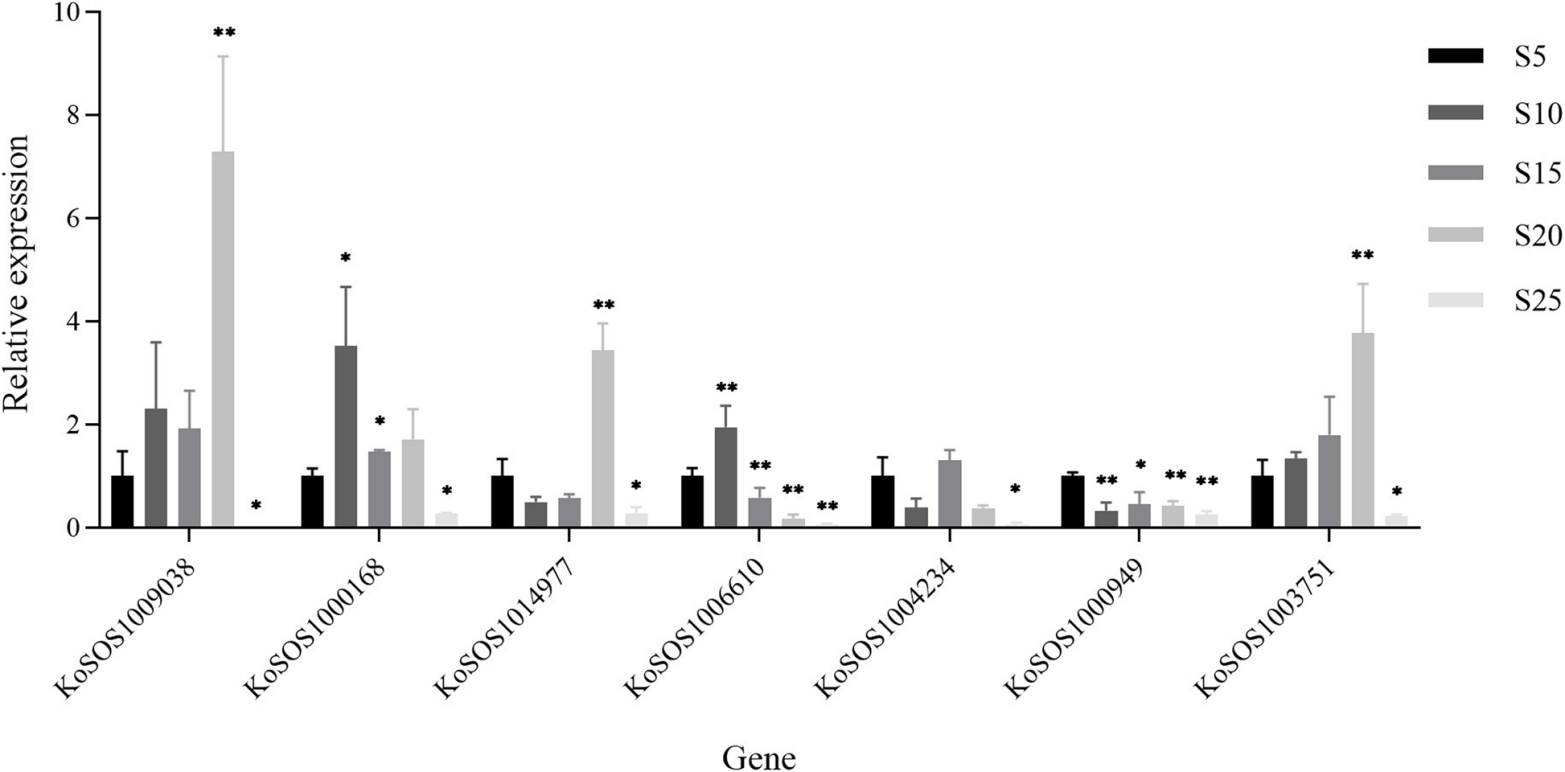
shows the results of the qRT-PCR study measuring *KoSOS1* expression in leaves of seedling-stage *Kandelia obovata* plants under different salt stress levels (S0, S5, S10, S15, and S20%). Using the Least Significant Difference (LSD) test, there is a significant difference (*p* < 0.05) between all conditions and the control group. An asterisk (*) is used to indicate the presence of significant discrepancies. A single asterisk (*) denotes a significance level of *p* < 0.05, whereas two asterisks (**) imply a significance level of *p* < 0.001. The vertical axis represents the relative gene expression, whereas the horizontal axis depicts the *KoSOS1* genes

We used qRT-PCR to examine the *SOS1* gene expression level in *Kandelia obovata* leaves under five different salt stress conditions (S0, S5, S10, S15, and S20%). A total of seven *SOS1* genes were examined: *KoSOS1009038*, *KoSOS000168*, *KoSOS1014977*, *KoSOS1006610*, *KoSOS1004234*, *KoSOS1000949*, and *KoSOS1003751* (Fig. 9). When compared to control (S5%), salt-stressed leaves (S20%) exhibited considerably higher (*p* < 0.05) expression levels of the *KoSOS1009038* gene. In S10% and S15%, the expression level of *KoSOS1009038* did not change (*p* > 0.05); however, in S25%, it did significantly decline (*p* < 0.05) in comparison to the control. The *KoSOS000168* gene was expressed at significantly greater (*p* < 0.05) levels in salt-stressed leaves (S10% and S15%) compared to the control. Compared to the control, *KoSOS000168's* expression level significantly decreased (*p* < 0.05) in S25% but did not change in S20% (*p* > 0.05). In response to the S20%, the *KoSOS1014977* gene showed statistically significant (*p* < 0.05) up-regulation in expression when compared to the control group (Cu0), but it remained unchanged (*p* > 0.05) in the S10% and S15%. In S25%, *KoSOS1014977* expression level considerably (*p* < 0.05) decreased compared to the control. When comparing salt-stressed leaves (S10%) to control, the expression of the *KoSOS1006610* gene was considerably higher (*p* < 0.05). The expression level of *KoSOS1006610* considerably decreased (*p* < 0.05) in S15%, S20%, and S25% treatment compared to the control. The expression level of the *KoSOS1004234* gene did not significantly alter (*p* > 0.05) in S5%, S10%, S15%, and S20% treatments, it did considerably decrease (*p* < 0.05) in S25% compared to the control. In comparison to the control, only one *KoSOS1000949* gene was expressed at significantly down-regulated (*p* < 0.05) levels in all salt-stressed leaves (S10%, S15%, S20%, and S25%). When the *KoSOS1003751* gene was compared to the control group (Cu0) in the S20%, there was a statistically significant (*p* < 0.05) up-regulation in expression; however, in the S10% and S15%, there was no change (*p* > 0.05). The expression level of *KoSOS1003751* in S25% was significantly (*p* < 0.05) lower than that of the control. When subjected to S25% salt stress, all *SOS1* genes demonstrated significant down-regulation, while *KoSOS1000949* showed significant down-regulation in all salt stress conditions (Fig. 9).

### Expression analysis of SOS1 genes *Kandelia obovata* leaves under copper stress

**Fig. 10:**
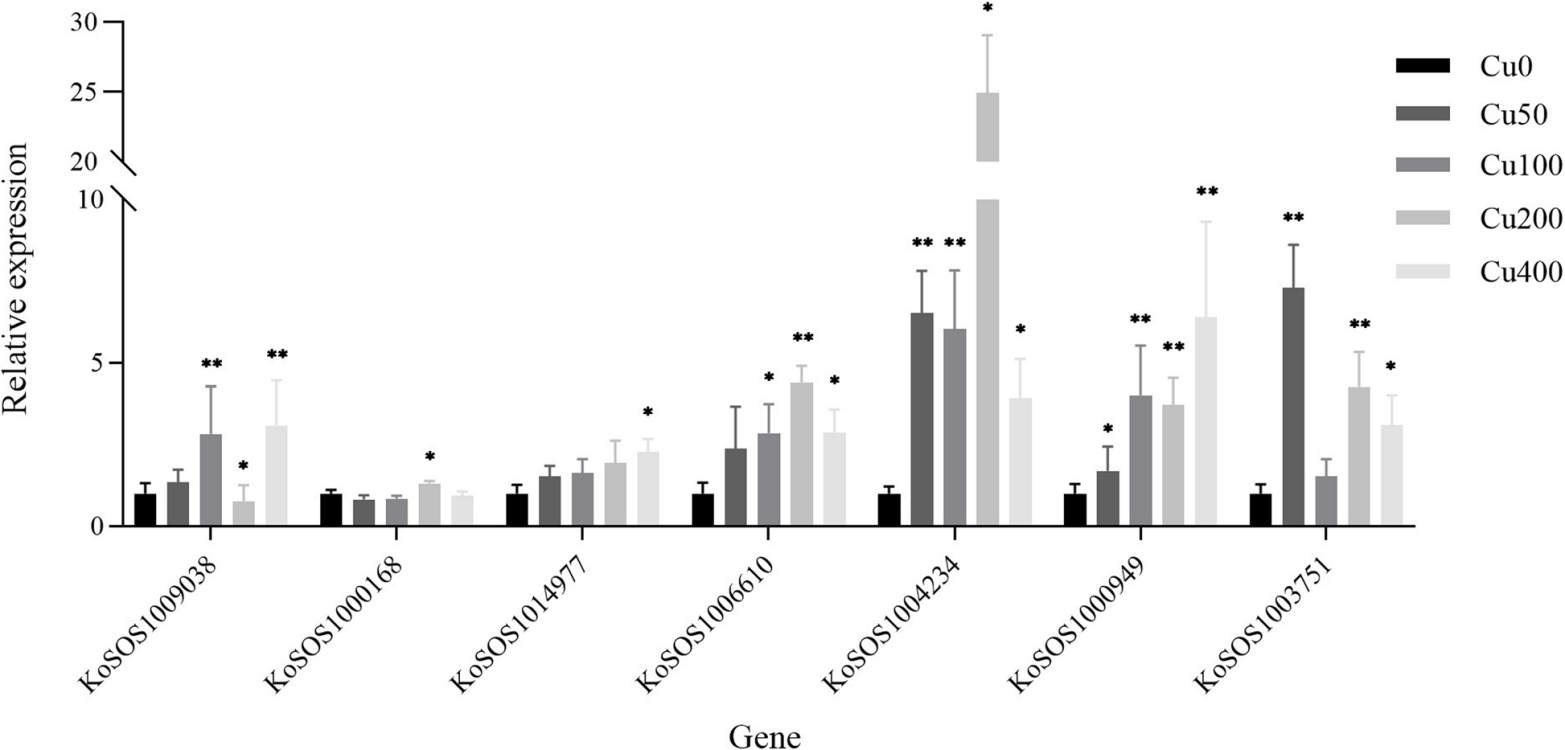
Using qRT-PCR, the expression of *KoSOS1s* was investigated in the leaves of seedling-stage *Kandelia obovata* plants under various copper stress conditions (Cu0, Cu50, Cu100, Cu200, and Cu400 mg L^−1^). The Least Significant Difference (LSD) test confirms a statistically significant difference (*p* < 0.05) between the control group and all treatment groups. An asterisk (*) is used to indicate the presence of significant differences, with * representing a significance level of *p* < 0.05 and ** representing a significance level of *p* < 0.001. The vertical axis represents the relative gene expression, whereas the horizontal axis depicts the *KoSOS1* genes

The expression level of the SOS1 gene in *Kandelia obovata* leaves was analyzed under five distinct copper stress conditions (Cu0, Cu50, Cu100, Cu200, and Cu400 mg L^−1^) using qRT-PCR. Seven SOS1 genes (*KoSOS1009038*, *KoSOS000168*, *KoSOS1014977*, *KoSOS1006610*, *KoSOS1004234*, *KoSOS1000949*, and *KoSOS1003751*) were investigated (Fig. 10). Expression levels of the *KoSOS1009038* gene were increased (*p* < 0.05) in copper-stressed leaves (Cu100 and Cu400) compared to control. The expression level of *KoSOS1009038* remained unchanged (*p* > 0.05) in Cu50, while in Cu200, the expression level decreased (*p* < 0.05) compared to the control. The expression levels of *KoSOS000168* and *KoSOS1014977* genes did not show statistical significance when comparing the *KoSOS000168* expression in Cu50, 100, and 400, while *KoSOS1014977* expression in Cu50, 100, and 200 with the control group. However, these genes exhibited statistically significant up-regulation in the Cu200 and Cu400 groups compared to the Cu0 group. The *KoSOS1006610* gene remained unchanged (*p* > 0.05) in Cu50, while it showed statistically significant (*p* < 0.05) up-regulation in expression in response to the Cu100, Cu200, and Cu400 treatment, as compared to the control group (Cu0). Only two genes, *KoSOS1004234* and *KoSOS1000949,* exhibited statistically significant up-regulation in the Cu50, Cu100, Cu200 and Cu400 groups compared to the Cu0 group. The expression level of the *KoSOS1003751* gene was up-regulated in Cu50, Cu200, and Cu400 but non-significant in Cu100 as compared to Cu0 treatment. Comparison among *KoSOS1* genes *KoSOS1009038* showed significant down-regulation, while *KoSOS1004234* and *KoSOS1000949* showed significant up-regulation under copper stress. Genes including *KoSOS1009038*, *KoSOS000168*, *KoSOS1014977*, *KoSOS1006610*, and *KoSOS1003751* showed no significant change in expression (Fig. 10).

When comparing the effects of salt and copper stress, it was observed that most genes exhibited reduced expression under all salt stress levels. The results indicate that nearly all of the *SOS1* genes exhibited considerable down-regulation under S25% salt stress. When comparing the *KoSOS1* genes, it was shown that *KoSOS1009038* exhibited a considerable decrease in expression, but *KoSOS1004234* and *KoSOS1000949* showed a large increase in expression in response to copper stress. *KoSOS1000949* exhibited considerable down-regulation under all salt stress conditions, while it displayed significant up-regulation under all copper stress levels.

## Discussion

Soil salinity is a major abiotic stress that crop plants in agricultural field’s worldwide encounter, resulting in a decrease in crop yield and production. Plants have developed the *SOS* pathway as a means to attain salt tolerance [20, 34]. The SOS pathway, which consists of *SOS1*, *SOS2*, and *SOS3,* is believed to regulate cellular signaling in response to salt stress in order to maintain ion balance [35]. *SOS1* has a crucial role in determining the resistance of plants to salt [22]. The *SOS1* genes have been documented to enhance salt stress resistance in various plant species, including potato [20], wheat [29], tuber mustard [21], Arabidopsis [6, 23], and quinoa [31]. Copper (Cu) is a vital element for all living species. The transport of Cu through the cell membrane is a critical step in maintaining Cu homeostasis [15]. Multiple transporter protein types have been reported to facilitate the absorption of copper [36]. Nevertheless, there is currently no information available regarding the response of the *SOS1* genes in the common woody mangrove species *Kandelia obovata* to copper-induced stress. In this study, we utilized genome-wide analysis to identify a reduced *SOS1* gene family consisting of 20 members from the genome of *Kandelia obovata*.

*Kandelia obovata* and *B. sexangula* are two types of mangrove species that belong to the *Rhizophoraceae* Pers. Family [37]. These species are known for their ability to exclude salt and are also related with the management of heavy metals [38–41]. *Kandelia obovata*, a type of woody plants, is primarily located in salt marshes throughout tropical and subtropical areas spanning from East Asia to Southeast Asia [41, 42]. *Kandelia obovata* successfully adapts to transitional habitats where the land and ocean meet, while facing both regular and irregular tidal forces. These influences lead to elevated salinity levels, significant erosion, and oxygen-deprived conditions [43]. Mangrove trees are tolerant to high salinity levels and can withstand considerable daily and yearly fluctuations in salinity. Gaining a comprehensive understanding of the molecular-level salinity tolerance mechanism in mangroves is crucial for the development of crops that can potentially be grown using the plentiful seawater [44]. Prior research has shown that mangrove seedlings can thrive in environments with exceptionally high levels of heavy metals [37, 38], and endure salt stress [45, 46], albeit with detrimental impacts on their growth. Mangroves possess a significant capacity to accumulate metals, enabling them to serve as a biogeochemical buffer for heavy metal contaminants. This capability has been demonstrated in several studies [37, 38, 40, 47]. This study aims to identify crucial *KoSOS1* genes that exhibit heightened sensitivity to abiotic stress. The objective is to establish a foundation for investigating the regulatory mechanisms of *SOS1* genes in *Kandelia obovata*.

The *KoSOS1* genes are crucial in plants for their response to salt and copper stress. In this study, a total of 20 proteins belonging to the *Kandelia obovata SOS1* family were classified into four distinct groups. The identification of six *SOS* genes, namely *SOS1*, *SOS2*, *SOS3*, *SOS4*, *SOS5*, and *SOS6*, was reported in Arabidopsis [23]. Plants that have been genetically modified to produce higher levels of *AtNHX1* or *SOS1* have shown a notable improvement in their ability to withstand high salt levels [23]. Researchers discovered a total of 37, 119, and 12 *SOS1* gene families in potato [20], wheat [29], and tuber mustard [21]. The SOS1 protein is produced by the *SOS1* gene and acts as a hypothetical plasma membrane Na^+^/H^+^ antiporter, responsible for removing Na^+^ ions from plant cells. Thus, it helps maintain the balance of K^+^ and Na^+^ levels in plant cells and prevents the buildup of Na^+^ in plant cells [20, 21, 29, 32]. Our work discovered a greater number of *SOS1* gene families in the *Kandelia obovata* genome, which is noteworthy. Our findings indicate that there are 20 *SOS1* homologs present in the genome of *Kandelia obovata*. The number of *SOS1* genes in the *Kandelia obovata* genome exceeds that in tuber mustard, primarily due to *Kandelia obovata* being an allopolyploid species.

El Mahi et al. [48] demonstrated that *SOS1* genes are crucial in facilitating plants' reactions to salt stress. Nevertheless, there has been no comprehensive examination conducted on the *SOS1* gene family in wheat, particularly regarding its expression patterns when subjected to salt stress. The chromosomal positions and collinearity suggest that segmental duplications are significant in the proliferation of *SOS1* members in wheat [29]. The phylogenetic analysis revealed that the *SOS1* genes obtained from *Kandelia obovata* and five additional plant species, specifically *Arabidopsis thaliana* (At), *Triticum aestivum* L. (Traes), *Solanum tuberosum* (St), *Oryza sativa* (Os), and *Bruguiera gymnorhiza* (Bg), were classified into four main groups. The current investigation entailed the discovery of 20 *SOS1* genes from the genome of *Kandelia obovata*. A phylogenetic analysis was performed, revealing that *KoSOS1s* and *Solanum tuberosum* (*StSOS1s*) exhibited the most closely related relationship [20].

Based on our subcellular localization prediction of SOS1 proteins, we identified 20 *KoSOS1s* that are most likely located in the plasma membrane. The subcellular localization of proteins has a crucial role in defining the function and accumulation patterns of plant proteins [49]. The expression of *CcSOS1* in *Chrysanthemum crassum* was seen in close proximity to the plasma membrane in onion epidermal cells that were temporarily converted [50]. The expression of *SOS1* genes in the plasma membrane was anticipated in potato [20], wheat [29], and tuber mustard [21]. The cis-elements and functional properties of SOS1 gene promoters have been found in many species, including potato [20], wheat [29], and tuber mustard [21]. In this study, we conducted an investigation of cis-acting regulatory elements in the promoter region of *SOS1s* in *Kandelia obovata* to gain a deeper understanding of their potential function. The cis-regulatory elements were discovered to encompass phytohormone and abiotic stressors, aligning with earlier research findings in other species [20].

Various investigations have confirmed that *KoSOS1s* play a role in numerous physiological reactions to salt and heavy metal exposure. The main aim of this work was to investigate the influence of salt and Cu stress on the expression of *KoSOS1*, a particular gene. The findings indicated that the expression levels of *KoSOS1* genes were seen to be both upregulated and downregulated in response to both salt and copper stress. Arabidopsis plants that have been genetically modified to have higher levels of the wheat *SOS1* gene have enhanced ability to withstand the negative effects of salt stress, as demonstrated in a study by Jiang et al. [29]. Prior research has demonstrated that *SOS1* is increased in response to salt stress in Arabidopsis [51]. The expression of SOS1 was markedly increased in leaf tissue during NaCl stress (450 mmol/L) [31]. It may be possible to improve crops' and plants' resistance to high salt and copper concentrations by using the *KoSOS1* genes as useful genetic modifiers. Some *SOS1* genes in potato showed both up-regulation and down-regulation in response to various stress conditions [20]. Comparably, in wheat, the transcriptome analysis showed that, under salinity stress, 28 and 26 genes, respectively, were up- and down-regulated; of these, 18 genes were further validated by RT-qPCR [29]. Similarly, in *Kandelia obovata*, *SOS1* genes were also found to be up-regulated and down-regulated in the presence of salt and copper stress.

## Conclusions

*Kandelia obovata's* genome has 20 *KoSOS1s*, according to this study's genome-wide examination of the *KoSOS1* genes. To learn more about the evolution of the *SOS1* gene family in the *Kandelia obovata* genome, various analyses were carried out, including gene identification, subcellular localization, chromosomal distributions, domain and 3D structural variation, phylogenetic tree, synteny and duplication analyses, gene structure, motif analysis, cis-regulatory elements, and expression profiling against different salt and copper treatments. *KoSOS1* genes were found on *Kandelia obovata's* 11 chromosomes. Four groups were identified by the phylogenetic analysis based on the *SOS1* proteins found in *Kandelia obovata*, *Arabidopsis thaliana*, *Triticum aestivum* L., *Solanum tuberosum*, *Oryza sativa*, and *Bruguiera gymnorhiza*. According to the expression profiles, most *KoSOS1* genes expressed themselves specifically in leaves and were mainly responsible for salt and copper resistance to stressful conditions. These results will also make identifying putative genes that improve plant architecture in response to stressors easier and pave the way for future *KoSOS1* gene breeding and genetic upgrades in other crops. These procedures may use CRISPR/Cas-mediated deletion, overexpression, and other genetic changes.

## Materials and Methods

### Identification and characterization of *SOS1* genes in *Kandelia obovata*

The NCBI database (https://www.ncbi.nlm.nih.gov/, BioProject/GWH, Accession codes: PRJCA002330/GWHACBH00000000) and the *Kandelia obovata* protein database (https://www.omicsclass.com/article/310) were used to acquire the genomic sequences for *Kandelia obovata* [42]. The hypothetical proteins were cross-referenced and validated using two databases: NCBI CDD (with an E-value of 1.2e-28) and Pfam (available at http://pfam.xfam.org/). The protein sequence analysis of *SOS1*, which is connected with the domain profile, was conducted using the Pfam database available at http://pfam.xfam.org. Twenty *SOS1* family genes were found and verified using the NCBI database (https://www.ncbi.nlm.nih.gov/) and the *Kandelia obovata* genome database (https://www.omicsclass.com/article/310) (Table S4, S5). Protparam (http://web.expasy.org/protparam/) was utilized to analyze the physicochemical properties.

### Chromosomal distribution of *SOS1* genes in *Kandelia obovata*

All of the *SOS1* genes in *Kandelia obovata* have their genomic locations and protein sequences determined using the NCBI database and https://www.omicsclass.com/article/310. We also examined the chromosomal distribution of SOS1 genes. The MapGene2Chromosome (MG2C) tool, available at http://mg2c.iask.in/mg2c v2.0/, was used to determine the chromosomal location of *SOS1* genes in *Kandelia obovata*.

### Phylogenetic tree construction

The protein sequences of *SOS1* genes from the following species were used in the phylogenetic analysis: *Kandelia obovata* (Ko), *Bruguiera gymnorhiza* (Bg), *Triticum aestivum* L. (Traes), Oryza sativa (Os), and *Solanum tuberosum* (St). The MEGA11 (V 6.06) software, available at www.megasoftware.net, was commonly utilized for protein sequence alignment. The phylogenetic tree was constructed using the neighbour-joining (NJ) method with 1000 bootstrap replicates. The phylogenetic tree was seen and modified using Fig Tree V1.4.4.

### Gene structure and significant motif analyses of the SOS1 family members

*Kandelia obovata's* genome contains a total of twenty (20) genes belonging to the *SOS1* family. Web software (http://gsds.cbi.pku.edu.cn) determined the structural analyses of 20 SOS1 genes and showed the exon/intron arrangements of the *SOS1* genes. Among the protein sequences of the 20 SOS1 proteins, the online program MEME v5.4.1, accessible at https://meme-suite.org/meme/tools/glam2scan, identified more conserved regions or groups. The program used the following settings: sequence alphabet (DNA, RNA, or protein); site distribution (zero or one occurrence per sequence; classic mode for motif searching), and 10 motifs. The MEME findings were displayed using the TBtools application after downloading the corresponding mast file.

### Synteny and duplication analysis

SOS1 gene synteny connections were determined in *Arabidopsis thaliana* (At), *Populus trichocarpa* (Pt), *Oryza sativa* (Os), and *Vitis vinifera* (Vv) using the Minspan method (available online). To investigate the evolutionary constraints of each *SOS1* gene pair, the synonymous (Ks), non-synonymous (Ka), and Ka/Ks ratios were calculated using the KaKs Calculator 2.0 (https://sourceforge.net/projects/kakscalculator2/).

### Analysis and prediction of cis-acting elements of the *SOS1* family

The *Kandelia obovata* genome assembly database was used to collect two thousand (2000 bp) upstream sequences of SOS1 family members. The PlantCARE tool (http://bioinformatics.psb.ugent.be/webtools/plantcare/html/) was utilized to detect cis-regulatory elements (CREs) in the obtained sequences. Figure 6 in TBtools was generated using the most prevalent cis-regulatory elements (CREs) identified for the *SOS1* genes based on the frequency count of each CRE motif.

### 3D structure and subcellular localization

SWISS-MODEL (https://swissmodel.expasy.org/interactive) can be used to estimate the three-dimensional (3D) structure.

Secondary structure refers to the local folding patterns of a protein or nucleic acid, precisely the arrangement of its amino acid or nucleotide residues. NPS@: SOPMA is a secondary structure prediction tool available at ibcp.fr.

Link to the transmembrane structure: https://services.healthtech.dtu.dk/services/TMHMM-2.0/

The subcellular location of the SOS1 family genes was predicted using two online tools.

(1) ProtComp 9.0 can be accessed at the following link: http://linux1.softberry.com/berry.phtml?topic=protcomppl&group=programs&subgroup=proloc

The user has entered the word "CELLO". Server: cello.life.nctu.edu.tw/.

### Plant material and environmental conditions

The research used one-year-old *Kandelia obovata* seedlings, three treatments, ten to twelve plants per treatment, and three replications. The seedlings were got and planted in the mangrove conservation site at Golden Bay Mangrove Reserve, which is located in Beihai, Guangxi Province, China. The site is located at the geographical coordinates of 109.22° N and 21.42° E. The soil was irrigated with CuCl_2_ solution and received regular morning and evening watering with seawater from the nearby area, as part of semi-natural agricultural techniques. Five different CuCl_2_ concentrations (0, 50, 100, 200, and 400 mg/L) were used for the treatments during two years. These concentrations are Cu0, Cu50, Cu100, Cu200, and Cu400. A starting concentration of 0 mg/L of Cu0 was used in the control treatment, which used local seawater. The soil sample utilized in this experiment had a Cu concentration below 1.0%, which was categorized as non-polluted [48]. The different salt concentrations were given twice a day after the transplanting, using either pure seawater or a combination of saltwater and sea salt to obtain the appropriate level of salinity. Seawater samples were taken near the shore as necessary between November 2017 and November 2019. From 2017 to 2018, the average salinity was recorded to be 19.74 ± 1.14% (*n* = 21). However, from 2019 to 2020, the average salinity increased to 22.12 ± 0.69% (*n* = 39). The concentrations of 5, 10, 15, 20, and 25% were represented by the five distinct salt levels—S5, S10, S15, S20, and S25. A starting concentration of S5% was used in the control treatment, which used local seawater. Plant samples were gathered to evaluate the various parameters after two years of salt and copper treatments. Our recently published paper Shang et al. [9] and Liao et al. [52] describes the precise procedures and quantification of soil characteristics, including the application of Cu and salt solution, evaluation of soil properties, and Cu and salt content in soil.

### Quantitative Real-Time PCR Assays

With the help of TRIzol (Invitrogen, http://www.invitrogen.com), total RNA was isolated from the leaves previously indicated. Quantitative real-time PCR (qRT-PCR) testing was carried out using the 2^−∆∆CT^ method on ABI PRISM 7500 Real-time PCR Systems from Applied Biosystems. Table 3 includes a comprehensive list of the exact *KoSOS1* gene primers used in this study. These primers were produced using the primerdesigning tool provided by the National Center for Biotechnology Information (NCBI) (https://www.ncbi.nlm.nih.gov/tools/primer-blast/. The actin gene (GWH-TACBH010383.1) was utilized as an internal control. Based on the sequence provided by Sun et al. [53], the reference gene for *Kandelia obovata* (KoActin) was chosen. Primers CAATGCAGCAGTTGAAGGAA and CTGCTGGAAGGAACCAAGAG were used as the forward and reverse primers, respectively. The statistical analysis was performed using GraphPad Prism 9.0.0, and the Student's t-test was conducted.

**Table 3:** Information about the primers used in this study's gene expression analysis by qRT-PCR

Gene Name	Primer Name	Sequence (5’-3’)	Length	Tm	GC%	Product Length
*KoSOS1007260.1*	1-F	CCGCTGTTAGTTCAACGCTGTT	22	58.1	50	172
	1-R	GCTATGGTAACCGCACCTCTCA	22	58.2	54.5	
*KoSOS1007616.1*	2-F	TATCGGCAGCCTCCAGCAGATT	22	59.9	54.5	103
	2-R	GAGCACGGCGAAGCAGAGAATT	22	59.9	54.5	
*KoSOS1009308*	3-F	TTAGGCGAGAGTTTTGAGGGG	21	59.72	52.38	260
	3-F	GACTAATCCTCGGTGACAGGG	21	59.52	52.38	
*KoSOS1000168*	4-F	TGCCTCATCCAAAGCAACCA	20	60.18	50	120
	4-R	GTAGAACAGTGTGCCCACCA	20	59.89	55	
*KoSOS1007241.1*	5-F	GCCGAGCACAACACTGAACAGA	22	59.7	54.5	146
	5-R	GGAGAGGCAACTGAGCACTGA	21	59.3	54.5	
*KoSOS1008568.1*	6-F	CCGCTGAGAGGTTGATGAGGAA	22	58.1	54.5	171
	6-R	CAACGGTTGTCTTCGAGCAAGT	22	57.8	50	
*KoSOS1014074.1*	7-F	GGATGTGCTTGACACCGAGGAA	22	58.9	54.5	150
	7-F	CCGTCAGCTTCAGCTCATACCA	22	58.5	54.5	
*KoSOS1014977*	8-F	CACGATCACAGAGCCAGTTCCT	22	58.4	54.5	204
	8-R	CTTCGGCACCAGACTCATCACT	22	58.4	54.5	
*KoActin*	F	CAATGCAGCAGTTGAAGGAA	20	62.1	45	
	R	CTGCTGGAAGGAACCAAGAG	20	63.4	55	

### Statistical analysis

The data was analyzed using one-way ANOVA in SPSS version 13.0. The results were shown as the mean SD (Standard Deviation) of the three replicates. Five different copper stress levels (Cu0, Cu50, Cu100, Cu200, and Cu400 mg L^−1^) and five different salt levels (S5, S10, S15, S20, and S25%) were compared for differences in leaf mean values using an LSD (least significant difference) test at *p* < 0.05. The statistical software GraphPad Prism 9 (https://www.graphpad.com; was used to make the graphs.

### Supplementary Information

Supplementary Material 1.

Supplementary Material 2.

Supplementary Material 3.

Supplementary Material 4.

Supplementary Material 5.

## Data Availability

Data pertaining to the study have been included in the article or as supplementary material, further inquiries can be directed to the corresponding author.

## References

[1] Hussain Q, Asim M, Zhang R, Khan R, Farooq S. Transcription Factors Interact with ABA through Gene Expression and Signaling Pathways to Mitigate Drought and Salinity Stress. Biomolecules. 2021;11:1159. 34439825 10.3390/biom11081159 PMC8393639

[2] He M, He CQ, Ding NZ. Abiotic stresses: General defenses of land plants and chances for engineering multistress tolerance. Front Plant Sci. 2018;871 December:1–18. 10.3389/fpls.2018.01771 PMC6292871 30581446

[3] Cramer GR, Urano K, Delrot S, Pezzotti M, Shinozaki K. Effects of abiotic stress on plants: a systems biology perspective. BMC Plant Biol. 2011;11:1–14. 22094046 10.1186/1471-2229-11-163 PMC3252258

[4] Waqas MA, Kaya C, Riaz A, Farooq M, Nawaz I, Wilkes A, et al. Potential Mechanisms of Abiotic Stress Tolerance in Crop Plants Induced by Thiourea. Front Plant Sci. 2019;10 October:1–14. 10.3389/fpls.2019.01336 PMC6828995 31736993

[5] Zhu JK. Abiotic Stress Signaling and Responses in Plants. Cell. 2016;167:313–24. 27716505 10.1016/j.cell.2016.08.029 PMC5104190

[6] Zhang Y, Zhou J, Ni X, Wang Q, Jia Y, Xu X, et al. Structural basis for the activity regulation of Salt Overly Sensitive 1 in Arabidopsis salt tolerance. Nat Plants. 2023;9:1915–23. 37884652 10.1038/s41477-023-01550-6

[7] Yang Y, Guo Y. Unraveling salt stress signaling in plants. J Integr Plant Biol. 2018;60:796–804. 29905393 10.1111/jipb.12689

[8] Zhu JK. Salt and drought stress signal transduction in plants. Annu Rev Plant Biol. 2002;53:247–73. 12221975 10.1146/annurev.arplant.53.091401.143329 PMC3128348

[9] Shang C, Chen J, Nkoh JN, Wang J, Chen S, Hu Z, et al. Biochemical and multi-omics analyses of response mechanisms of rhizobacteria to long-term copper and salt stress: Effect on soil physicochemical properties and growth of *Avicennia marina*. J Hazard Mater. 2024;466 January:133601. 10.1016/j.jhazmat.2024.133601 38309159

[10] Sudhir S, Arunprasath A, Sankara Vel V. A critical review on adaptations, and biological activities of the mangroves. J Nat Pestic Res. 2022;1 February:100006.

[11] Alhassan AB, Aljahdali MO. Sediment Metal Contamination, Bioavailability, and Oxidative Stress Response in Mangrove *Avicennia marina* in Central Red Sea. Front Environ Sci. 2021;9 June:1–15.

[12] Yuan M, Li X, Xiao J, Wang S. Molecular and functional analyses of *COPT/Ctr*-type copper transporter-like gene family in rice. BMC Plant Biol. 2011;11:69. 21510855 10.1186/1471-2229-11-69 PMC3103425

[13] Hu Z, Fu Q, Zheng J, Zhang A, Wang H. Transcriptomic and metabolomic analyses reveal that melatonin promotes melon root development under copper stress by inhibiting jasmonic acid biosynthesis. Hortic Res. 2020;7:79. 32528691 10.1038/s41438-020-0293-5 PMC7261800

[14] Cano-Gauci DF, Sarkar B. Reversible zinc exchange between metallothionein and the estrogen receptor zinc finger. FEBS Lett. 1996;386:1–4. 8635592 10.1016/0014-5793(96)00356-0

[15] Hussain Q, Ye T, Li S, Nkoh JN, Zhou Q, Shang C. Genome-Wide Identification and Expression Analysis of the Copper Transporter (*COPT/Ctr*) Gene Family in *Kandelia obovata*, a Typical Mangrove Plant. Int J Mol Sci. 2023;24:15579. 37958561 10.3390/ijms242115579 PMC10648262

[16] Peñarrubia L, Andrés-Colás N, Moreno J, Puig S. Regulation of copper transport in *Arabidopsis thaliana*: A biochemical oscillator? J Biol Inorg Chem. 2010;15:29–36. 19798519 10.1007/s00775-009-0591-8

[17] Burkhead JL, Gogolin Reynolds KA, Abdel-Ghany SE, Cohu CM, Pilon M. Copper homeostasis. New Phytol. 2009;182:799–816. 19402880 10.1111/j.1469-8137.2009.02846.x

[18] Shen X, Li R, Chai M, Cheng S, Niu Z, Qiu GY. Interactive effects of single, binary and trinary trace metals (lead, zinc and copper) on the physiological responses of *Kandelia obovata* seedlings. Environ Geochem Health. 2019;41:135–48. 29987496 10.1007/s10653-018-0142-8

[19] Hussain Q, Ye T, Shang C, Li S, Nkoh JN, Li W, et al. Genome-Wide Identification, Characterization, and Expression Analysis of the Copper-Containing Amine Oxidase Gene Family in Mangrove *Kandelia obovata*. Int J Mol Sci. 2023;24:17312. 38139139 10.3390/ijms242417312 PMC10743698

[20] Liang L, Guo L, Zhai Y, Hou Z, Wu W, Zhang X, et al. Genome-wide characterization of *SOS1* gene family in potato (*Solanum tuberosum*) and expression analyses under salt and hormone stress. Front Plant Sci. 2023;14 June:1201730. 10.3389/fpls.2023.1201730 PMC10347410 37457336

[21] Cheng C, Zhong Y, Wang Q, Cai Z, Wang D, Li C. Genome-wide identification and gene expression analysis of *SOS* family genes in tuber mustard (*Brassica juncea var. Tumida)*. PLoS One. 2019;14:1–19. 10.1371/journal.pone.0224672 PMC6844470 31710609

[22] Świeżawska B, Duszyn M, Jaworski K, Szmidt-Jaworska A. Downstream targets of cyclic nucleotides in plants. Front Plant Sci. 2018;9 October:1–7. 10.3389/fpls.2018.01428 PMC6174285 30327660

[23] Yang Q, Chen ZZ, Zhou XF, Yin HB, Li X, Xin XF, et al. Overexpression of *SOS* (salt overly sensitive) genes increases salt tolerance in transgenic Arabidopsis. Mol Plant. 2009;2:22–31. 19529826 10.1093/mp/ssn058 PMC2639737

[24] Keisham M, Mukherjee S, Bhatla SC. Mechanisms of sodium transport in plants—Progresses and challenges. Int J Mol Sci. 2018;19:647. 29495332 10.3390/ijms19030647 PMC5877508

[25] Zhao C, William D, Sandhu D. Isolation and characterization of Salt Overly Sensitive family genes in spinach. Physiol Plant. 2021;171:520–32. 32418228 10.1111/ppl.13125

[26] Shi H, Ishitani M, Kim C, Zhu JK. The Arabidopsis thaliana salt tolerance gene *SOS1* encodes a putative Na^+^/H^+^ antiporter. Proc Natl Acad Sci U S A. 2000;97:6896–901. 10823923 10.1073/pnas.120170197 PMC18772

[27] Gao J, Sun J, Cao P, Ren L, Liu C, Chen S, et al. Variation in tissue Na+ content and the activity of *SOS1* genes among two species and two related genera of Chrysanthemum. BMC Plant Biol. 2016;16:98. 27098270 10.1186/s12870-016-0781-9 PMC4839091

[28] Núñez-Ramírez R, Sánchez-Barrena MJO, Villalta I, Vega JF, Pardo JM, Quintero FJ, et al. Structural insights on the plant salt-overly-sensitive 1 (*SOS1*) Na^+/^H^+^ antiporter. J Mol Biol. 2012;424:283–94. 23022605 10.1016/j.jmb.2012.09.015

[29] Jiang W, Pan R, Buitrago S, Wu C, Abou-Elwafa SF, Xu Y, et al. Conservation and divergence of the *TaSOS1* gene family in salt stress response in wheat (*Triticum aestivum* L.). Physiol Mol Biol Plants. 2021;27:1245–60. 10.1007/s12298-021-01009-y PMC8212347 34177146

[30] Cao Y, Shan T, Fang H, Sun K, Shi W, Tang B, et al. Genome-wide analysis reveals the spatiotemporal expression patterns of *SOS3* genes in the maize B73 genome in response to salt stress. BMC Genomics. 2022;23:1–13. 35034642 10.1186/s12864-021-08287-6 PMC8761280

[31] Maughan PJ, Turner TB, Coleman CE, Elzinga DB, Jellen EN, Morales JA, et al. Characterization of Salt Overly Sensitive 1 (*SOS1*) gene homoeologs in quinoa (*Chenopodium quinoa Willd.*). Genome. 2009;52:647–57. 10.1139/G09-041 19767895

[32] Zhang M, Cao J, Zhang T, Xu T, Yang L, Li X, et al. A Putative Plasma Membrane Na^+^/H^+^ Antiporter *GmSOS1* Is Critical for Salt Stress Tolerance in *Glycine max*. Front Plant Sci. 2022;13 May:870695. 10.3389/fpls.2022.870695 PMC9149370 35651772

[33] Zhou X, Li J, Wang Y, Liang X, Zhang M, Lu M, et al. The classical *SOS* pathway confers natural variation of salt tolerance in maize. New Phytol. 2022;236:479–94. 35633114 10.1111/nph.18278

[34] Cha JY, Kim J, Jeong SY, Shin GI, Ji MG, Hwang JW, et al. The Na^+^/H^+^ antiporter SALT OVERLY SENSITIVE 1 regulates salt compensation of circadian rhythms by stabilizing GIGANTEA in Arabidopsis. Proc Natl Acad Sci U S A. 2022;119: e2207275119. 35939685 10.1073/pnas.2207275119 PMC9388102

[35] Luo B, Guang M, Yun W, Ding S, Ren S, Gao H. *Camellia sinensis* Chloroplast Fluoride Efflux Gene CsABCB9 Is Involved in the Fluoride Tolerance Mechanism. Int J Mol Sci. 2022;23:7756. 35887104 10.3390/ijms23147756 PMC9317437

[36] Hussain Q, Ye T, Shang C, Li S, Mustafa AEMA, Elshikh MS. NRAMP gene family in Kandelia obovata: genome-wide identification, expression analysis, and response to five different copper stress conditions. Front Plant Sci. 2024;14:1318383. 38239217 10.3389/fpls.2023.1318383 PMC10794735

[37] Ma L, Yang S. Growth and physiological response of *Kandelia obovata* and *Bruguiera sexangula* seedlings to aluminum stress. Environ Sci Pollut Res. 2022;29:43251–66. 10.1007/s11356-021-17926-0 PMC9148292 35091926

[38] Dai M, Lu H, Liu W, Jia H, Hong H, Liu J, et al. Phosphorus mediation of cadmium stress in two mangrove seedlings *Avicennia marina* and *Kandelia obovata* differing in cadmium accumulation. Ecotoxicol Environ Saf. 2017;139 January:272–9. 10.1016/j.ecoenv.2017.01.017 28161586

[39] Zhang F, Wang Y, Lou Z. Effect of heavy metal stress on antioxidative enzymes and lipidperoxidation in leaves and roots of two mangrove plant seedlings (*Kandelia candel* and *Bruguiera gymnorrhiza*). Chemosphere. 2007;67:44–50. 17123580 10.1016/j.chemosphere.2006.10.007

[40] MacFarlane GR, Koller CE, Blomberg SP. Accumulation and partitioning of heavy metals in mangroves: A synthesis of field-based studies. Chemosphere. 2007;69:1454–64. 17560628 10.1016/j.chemosphere.2007.04.059

[41] Hu MJ, Sun WH, Tsai WC, Xiang S, Lai XK, Chen DQ, et al. Chromosome-scale assembly of the *Kandelia obovata* genome. Hortic Res. 2020;7:75. 32377365 10.1038/s41438-020-0300-x PMC7195387

[42] Sheue CR, Liu HY, Yong JWH. *Kandelia obovata* (Rhizophoraceae), a new mangrove species from Eastern Asia. Taxon. 2003;52:287–94. 10.2307/3647398

[43] Wang CW, Wong SL, Liao TS, Weng JH, Chen MN, Huang MY, et al. Photosynthesis in response to salinity and submergence in two Rhizophoraceae mangroves adapted to different tidal elevations. Tree Physiol. 2022;42:1016–28. 34918132 10.1093/treephys/tpab167

[44] Natarajan P, Murugesan AK, Govindan G, Gopalakrishnan A, Kumar R, Duraisamy P, et al. A reference-grade genome identifies salt-tolerance genes from the salt-secreting mangrove species *Avicennia marina*. Commun Biol. 2021;4:851. 34239036 10.1038/s42003-021-02384-8 PMC8266904

[45] Wang HM, Xiao XR, Yang MY, Gao ZL, Zang J, Fu XM, et al. Effects of salt stress on antioxidant defense system in the root of *Kandelia candel*. Bot Stud. 2014;55:55–7. 28510976 10.1186/s40529-014-0057-3 PMC5430347

[46] Xing J, Pan D, Wang L, Tan F, Chen W. Proteomic and physiological responses in mangrove *kandelia candel* roots under short-term high-salinity stress. Turkish J Biol. 2019;43:314–25. 10.3906/biy-1906-22 PMC6823913 31768104

[47] Zhou Y wu, Zhao B, Peng Y sheng, Chen G zhu. Influence of mangrove reforestation on heavy metal accumulation and speciation in intertidal sediments. Mar Pollut Bull. 2010;60:1319–24. 10.1016/j.marpolbul.2010.03.010 20378130

[48] Mahi H El, Pérez-hormaeche J, Luca A De, Villalta I, Espartero J, Gámez-arjona F, et al. A Critical Role of Sodium Flux via the Plasma Membrane Na^+^/H^+^ Exchanger *SOS1* in the Salt Tolerance. Plant Physiol. 2019;180 June:1046–65. 10.1104/pp.19.00324 PMC6548274 30992336

[49] Castleden IR, Aryamanesh N, Black K, Grasso SV, Millar AH. CropPAL for discovering divergence in protein subcellular location in crops to support strategies for molecular crop breeding. Plant J. 2020;104:812–27. 32780488 10.1111/tpj.14961

[50] Song A, Lu J, Jiang J, Chen S, Guan Z, Fang W, et al. Isolation and characterisation of *Chrysanthemum crassum SOS1*, encoding a putative plasma membrane Na^+^⁄H^+^ antiporter. Plant Biol. 2012;14:706–13. 22404736 10.1111/j.1438-8677.2011.00560.x

[51] Donaldson L, Ludidi N, Knight MR, Gehring C, Denby K. Salt and osmotic stress cause rapid increases in *Arabidopsis thaliana* cGMP levels. FEBS Lett. 2004;569:317–20. 15225654 10.1016/j.febslet.2004.06.016

[52] Liao J, Xu Y, Zhang Z, Zeng L, Qiao Y, Guo Z, et al. Effect of Cu addition on sedimentary bacterial community structure and heavy metal resistance gene abundance in mangrove wetlands. Front Mar Sci. 2023;10 April:1157905.

[53] Sun MM, Liu X, Huang XJ, Yang JJ, Qin PT, Zhou H, Jiang MG, Liao HZ. Genome-Wide Identification and Expression Analysis of the *NAC* Gene Family in *Kandelia Obovata*, a Typical Mangrove Plant. Curr Issues Mol Biol. 2022;44:5622–37. 36421665 10.3390/cimb44110381 PMC9689236

